# Influence of multiplicative stochastic variation on translational elongation rates

**DOI:** 10.1371/journal.pone.0191152

**Published:** 2018-01-19

**Authors:** Sandip Datta, Brian Seed

**Affiliations:** Center for Computational and Integrative Biology, Massachusetts General Hospital, Boston, United States of America; University of Edinburgh, UNITED KINGDOM

## Abstract

Experimental data indicate that stochastic effects exerted at the level of translation contribute substantially to the variation in abundance of proteins expressed at moderate to high levels. This study analyzes the theoretical consequences of fluctuations in residue-specific elongation rates during translation. A simple analytical framework shows that rate variation during elongation gives rise to protein production rates that consist of sums of products of random variables. Simulations show that because the contribution to total variation of products of random variables greatly exceeds that of sums of random variables, the overall distribution exhibits approximately log-normal behavior. Empirical fits of the data can be satisfied by either sums of log-normal distributions, or sums of log-normal and log-logistic distributions. Elongation rate stochastic variation offers an accounting for a major component of biological variation. The analysis provided here highlights a probability distribution that is a natural extension of the Poisson and has broad applicability to many types of multiplicative noise processes.

## Introduction

Regulation of the abundance of proteins expressed in living cells is mediated by multiple types of control, exerted over the rates of transcription, post-transcriptional mRNA processing, mRNA decay, translation, and protein degradation. Regulatory variation can give rise to fluctuations in protein concentration in an otherwise homogeneous cell population at steady state [1]. Stochastic fluctuations in protein distribution can result in heterogeneous phenotypes in clonal populations that can be beneficial for the survival of a population of organisms in a changing environment [2]. Task-sharing decisions assisted by stochastic differentiation in a clonal population may have formed the basis for multi-cellular development [2, 3]. In mammalian cells, manifestations of stochastic gene expression resulting in phenotypic diversity has been observed in the processes of cellular differentiation [2] and apoptosis [4].

The correlation between mRNA and protein abundances (or lack thereof) can be used as a guide to the extent by which fluctuations in mRNA copy number produce fluctuations in protein concentration [5]. Previous studies have found that the correlation between mRNA and protein levels is poor across all organisms [6, 7]. These studies have broadly indicated that post-transcriptional effects determine steady state protein abundance [8]. For example the simultaneous measurement of mRNA and protein abundance for 5000 genes in mouse fibroblasts revealed that about 55% of the correlation between mRNA and protein level can be explained by variation in translation rate [7]. These findings are consistent with conclusions reached in earlier studies that among post-transcriptional steps, variation in translational rate is a consequential stochastic factor for determining variation in protein abundance in the cell as a whole [1, 9]. Cells expend more energy in translation compared to transcription (in an approximately 9:1 ratio), which may explain the dominance of translational control.

The contribution of elongation to translation rate has been reviewed recently [10]. Initiation can be rate limiting for translation under some circumstances, but for the majority of moderately to highly expressed proteins, elongation is rate limiting for protein synthesis [11]. Among many cases that exemplify the rate limiting role of elongation [10], one of the the more compelling is the widespread applicability of codon optimization to improve protein production. Codon optimization methods systematically replace naturally occurring codons with synonymous codons corresponding to the most prevalent tRNAs for each amino acid to increase the production of proteins by increasing the rate of elongation [12, 13]. In both prokaryotic [14] and eukaryotic [15] contexts it is well known that high level expression of gene products requires codon optimization, and codon optimization is routinely applied in nearly all industrial gene expression systems. Appendix C provides a simple heuristic exposition on codon optimization and translational rate limitation.

The analysis described here is directed at exploring the stochastic consequences of variation in individual rates of elongation by ribosomes transiting an mRNA. The general mathematical framework has been explored in considerable detail, but with a different objective, by Gilchrist and Wagner, who have studied the consequences of premature termination and of ribosome recycling [16]. Among the more directly relevant conclusions of their work is that translation can be treated as a stochastic progression through a heterogeneous medium, a premise that plays a central role in the model and conclusions presented here.

Another highly studied model is the non-equilibrium totally asymmetric exclusion process (TASEP) [17–19], which shares some features with the model presented here. Common to both treatments is the assumption that initiation is not rate-limiting, a prerequisite for ribosomes to undergo interaction. The focus of the present work, however, is an accounting for log-normality in biological systems. The TASEP model has been used to study many aspects of translation including the effect of ribosome recycling [17] and also to highlight the role of slow codons in creating clustered bottlenecks [18].

The approximately log-normal character of protein abundance distributions is often demonstrated by quantitative flow cytometry, in which the fluorescence intensity of fluorophore labeled proteins are determined on a cell-by-cell basis. As conventionally displayed in logarithmic coordinates, the number of fluorophores per cell typically exhibits a more or less normal density profile. These observations have been corroborated by results from quantitative mass spectrometry of protein fragments, which also show an approximately log-normal probability density [20]. Various other methods for protein quantitation have led to similar conclusions [20–22]. Nearly all microarray analyses use statistical tests based on the logarithm of raw transcript abundance data [23], consistent with single cell gene expression measurements indicating that mRNA abundance distributions are log-normally distributed in cell populations [24, 25]. In the case of low-abundance proteins the protein abundances have been shown to be reasonably well fit to the gamma distribution but one cautionary note is that extreme outliers are often seen in published data sets, consistent with the influence of unrecognized multiplicative noise [26–28].

The exposition here is divided in sections as follows. Section II discusses the noise characteristics at various levels of protein abundances. Section III shows that the elongation phase of translation proceeds at a variable rate for any particular mRNA in a population of cells. Section IV describes the stochastic model used for modeling translation. The simulation results describing the distribution of proteins in the steady state are discussed in Section V. Section V gives the distribution of the number of polypeptides per transcript. Section VI describes the dynamics the transient state, and Section VII presents concluding remarks. Appendix A gives the detailed derivations of the analytical results presented in Sections III and IV. Appendix B presents a note on the numerical simulations, and Appendix C is a table of symbols.

## Characterizing noise in translation

Elowitz et. al. have distinguished intrinsic and extrinsic sources of stochastic noise in biological systems [29]. For intrinsic sources the resulting noise (normalized by the squared mean abundance) is inversely proportional to the mean protein abundance [30], and deviations from this relation help to identify the relative proportion of extrinsic noise. Xie and coworkers have carried out single molecule measurements in *E. coli* cells under conditions in which expression is highly repressed, so that intrinsic noise due to random formation and degradation of RNA molecules is the dominant noise source [26, 27]. They found that, below ten proteins per cell the noise is inversely proportional to protein abundance [28]. They also showed that the distribution of such low copy number proteins can be fit to a gamma distribution, the two parameters of which have direct physical interpretation as the protein burst rate and burst size [28, 31]. This direct physical interpretation of parameters is lost, however, above ten proteins per cell when the noise reaches a plateau indicating the dominance of extrinsic noise [28, 31] (Reported numbers of proteins per bacterial cell can range from approximately 50,000 to nearly zero [32, 33]).

Detailed experimental measurements carried out in *S. cerevisiae* have shown that the contribution of extrinsic noise to protein abundance increases with level of expression [1, 34]. Factors such as ribosome number fluctuations and variations in kinetic parameters like elongation rates, have been categorized as extrinsic noise [1, 9].

## Elongation proceeds at a variable rate

Translation can be divided into four stages: initiation, elongation, termination and recycling. The mechanisms for initiation and termination differ between prokaryotes and eukaryotes, but the elongation mechanism is conserved [35]. In the elongation phase, a series of reactions leads to the addition of amino acid residues to the polypeptide chain (or rejection of the aa-tRNA), and each of these reactions can be characterized in terms of kinetic rate constants [36]. As a simplification, a net effective rate constant for a single residue addition in elongation can be composed from the rate constants for individual steps that result in chain extension. The effective kinetic rate constant is expected to fluctuate from cell to cell across a cell population, depending on a variety of noise sources, the vast majority of which will arise from proteins that compose the translational machinery and are expressed at a high level and hence are expected to contribute extrinsic noise.

The rates at which elongation proceeds vary widely; for example in bacteria the elongation rate varies between 4 and 22 amino acids per second [11]. The protein synthesis rate can be affected by many factors, of which the most significant is considered to be the relative concentration of various tRNAs [14, 37]. At each elongation step the ribosome must intercept the aminoacyl-tRNA (aa-tRNA) complementary to the codon at the ribosome A site. The relative local concentration of various aa-tRNAs near the site determines the waiting time. Codons corresponding to underrepresented tRNAs reduce the elongation rate [37, 38] and are themselves underrepresented [39], which is thought to represent a mechanism allowing organisms to manipulate the expression level of proteins [40]. Elongation is also slowed by mRNA secondary structures called pseudoknots [41] or by the interaction of nascent peptide sequences with the ribosome exit channel [42]. Sequencing of ribosome-protected mRNA fragments has provided a detailed picture of the ribosome distribution on mRNA [43]. A substantial variation in the density of ribosome footprints can be found for mRNAs from both yeast and *E. coli.* In mammalian cells, some mRNA locations have been found to have 25-fold greater density than the median density across the gene [43]. Similar translational pauses have been reported in other studies [44, 45]. Additional factors affecting elongation rate included collisions between individual ribosomes in polysomes [46], controlled ribosome stalling, and interactions between the translating ribosome and RNA polymerase in prokaryotes [47].

## A stochastic model for the translation process

**Fig 1:**
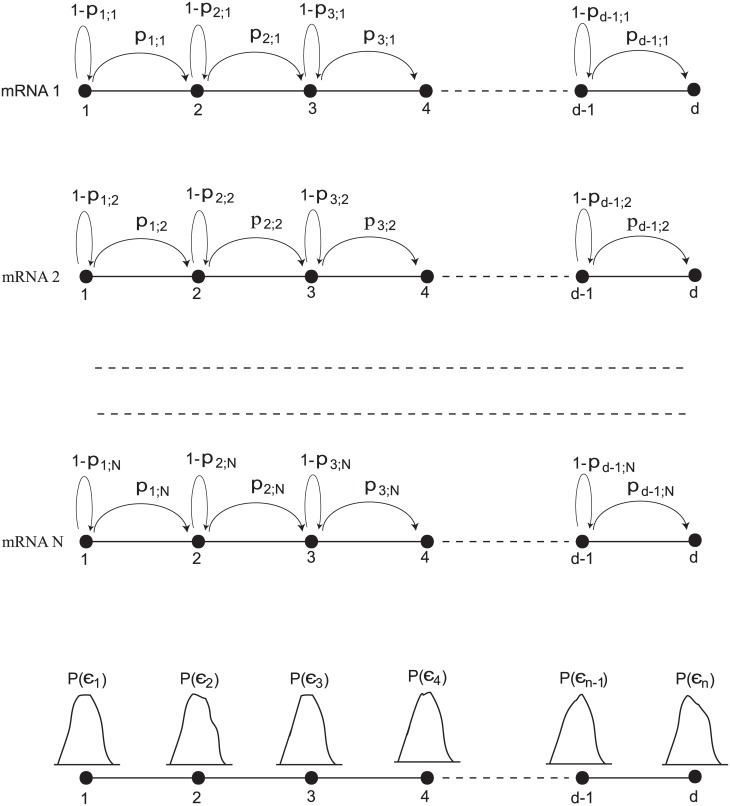
Schematic diagram of the stochastic random walk model for the translation of mRNAs. Following initiation the translation proceeds via elongation at different local rates on different mRNAs. The figure shows N copies of mRNA in a population of *N*_*c*_ cells undergoing elongation. Each mRNA is represented by a linear discrete lattice with individual nodes representing codons at which ribosomes add residues to the nascent polypeptide chain. At any given node *i* in an infinitesimal interval of time, the probability of a codon being translated or not is given by *p*_*i*_ and 1 − *p*_*i*_ respectively. The distribution of *p*_*i*_ at the *i*th node is given by *ρ*(*p*_*i*_). *p*_*i*;*j*_ represents the scaled rate constant (drawn from probability distribution *ρ*(*p*_*i*_)) for jth mRNA at the *i*th site. Once the ribosome reaches site *n*, the termination site, a completed protein is released and the ribosome moves back to the initiation site (the recycling step, found in eukaryotic cells.)

A significant fraction of the elongation rate variability is codon-specific, resulting in the translation of different codons at different rates [36, 48]. Gromadski and Rodnina measured the rate constants for different kinetic substeps for the CUC codon [48]. For any particular mRNA in a collection of cells, the rate constant at any arbitrary location along the mRNA is a stochastic variable and therefore can be described most appropriately by a distribution. With this consideration, the stochastic movements of a set (ensemble) of ribosomes along the mRNA during elongation under the collective influence of various noise sources can be modeled as a unidirectional random walk on a chain with circular boundary conditions (see Fig 1), with each incorporation of a residue taken to follow first order kinetics, with rate constant *ϵ*_*i*_ for the *i*th residue.

The circular boundary condition is applied as a general analysis of elongation rates should take into account the propensity for eukaryotic ribosomes to undergo reinitiation following completion of translation, a phenomenon that is physically visualized under conditions of high protein synthesis as a circular template structure [49, 50]. Ribosome recycling is now established as an integral stage of the translation process [16, 51, 52]. In general translation on a linear template that persists for a period of time sufficient to allow a ribosome to transit the entire reading frame can be modeled with no loss of generality as a circular lattice, by the simple device of adding a new codon “0” representing the free ribosome pool. That is, a ribosome transits from the termination codon to codon 0 to the initiation codon (Appendix C).

An individual mRNA is represented by a discrete lattice, the nodes of which represent codons. At any arbitrary node *i* in a discrete fixed period of time, the probability of a codon being translated or not is given by *p*_*i*_ and 1 − *p*_*i*_ respectively. The probability density of *p*_*i*_ at the *i*th node is given by an unknown *ρ*(*p*_*i*_). *ϵ*_*i*;*j*_ represents the scaled rate constant (drawn from probability distribution *ρ*(*p*_*i*_)) for *j*th mRNA at the *i*th site. Because the distribution of tRNAs in the environment of each mRNA is likely to be slightly different from one transcript to the next, a conservative assumption is that rates at a given position vary from template to template. When the ribosome reaches the termination site *d*, a completed protein is released and the ribosome returns to the initiation site (recycling step in eukaryotic cells) or the free ribosome pool. In the eukaryotic model a reinitiation probability, λ, conveys the likelihood of reinitiation once a ribosome has reached the end of the open reading frame of *d* codons.

To make the transition from a matrix of discrete probabilities to a stochastic process we follow the usual prescription for the identification of an appropriate generator of the convolution semi-group, e.g [53]. The time evolution can be described as a compound Poisson process, in which the evolution of the ribosome motion for given initial conditions is determined by the exponentiation of the generator. The lapse of an interval of time, *t*, results in a change in the probability state vector *V*_*i*_(*t*) = *Q*_*ij*_(*t*)*V*_*j*_(0) for the leading ribosome position determined by the initial conditions and

$$\begin{array}{c} Q\left(t\right)=e^{-\alpha t}\sum_{k=0}^{\infty }\frac{{\left(\alpha t\right)}^{k}}{k!}U^{k}=e^{\alpha t(U-I)} \end{array}$$ (1)

where *U* is a stationary transition matrix of the length of the polypeptide, *d*, having generator *T* = *U* − *I* where *I* is the unit matrix of length *d*. *α* is a scaling/normalization factor with dimension reciprocal time that converts the transition probabilities *p*_*i*_ into rate constants *ϵ*_*i*_, chosen here so that *α* *p*_*i*_ = *ϵ*_*i*_. The transition matrix (see Fig 1) describing movement of codon along the length of the mRNA which produces a polypeptide of length *d*, is given by

$$\begin{array}{c} U=(\begin{array}{ccccc} 1-p_{1} & 0 & \cdots & 0 & \lambda \;p_{d}\\ p_{1} & 1-p_{2} & \cdots & 0 & 0\\ \vdots & \vdots & \ddots & \vdots & \vdots \\ 0 & 0 & \cdots & 1-p_{d-1} & 0\\ 0 & 0 & \cdots & p_{d-1} & 1-p_{d} \end{array}) \end{array}$$ (2)

For the circular boundary conditions characteristic of eukaryotic elongation, the *i*, *j* entry of the exponentiation of this matrix for *i* < *j* yields (see Appendix A)

$$\begin{array}{c} Q{\left(t\right)}_{i,j}=\frac{1}{2\pi ı}\oint \frac{e^{ts}\lambda \prod_{k=i+1}^{j-1}\left(s+\epsilon_{k}\right)\prod_{m=j}^{d}\epsilon_{m}\prod_{m=1}^{i-1}\epsilon_{m}}{\prod_{k=1}^{d}\left(s+\epsilon_{k}\right)-\lambda \prod_{m+1}^{d}\epsilon_{m}}ds \end{array}$$ (3)

with $ı=\sqrt{-1}$. For *i* ≥ *j* Eq (3) yields

$$\begin{array}{c} Q{\left(t\right)}_{i,j}=\frac{1}{2\pi ı}\oint \frac{e^{ts}\prod_{k=1}^{j-1}\left(s+\epsilon_{k}\right)\prod_{k=i+1}^{d}\left(s+\epsilon_{k}\right)\prod_{m=j}^{i-1}\epsilon_{m}}{\prod_{k=1}^{d}\left(s+\epsilon_{k}\right)-\lambda \prod_{m+1}^{d}\epsilon_{m}}ds \end{array}$$ (4)

evaluated so that the contour encircles all the poles of the integrand, or, equivalently, encircles the pole at infinity in the opposite sense. For large *d* the product $\prod_{m=1}^{d}\epsilon_{m}$ is close to zero, and hence the roots of the denominator polynomial are expected to lie in the vicinity of *s* = −*ϵ*_*k*_.

This picture is simplified in the case of short lived mRNAs or in the prokaryotic context, in which the contribution of ribosome recycling can be ignored. Setting λ = 0 in Eqs (3) and (4) the elements of exponentiated matrix above reduce to

$$\begin{array}{l} Q{\left(t\right)}_{i,j}=\\ \{\begin{array}{cc} 0 & i<j\\ {\left(-1\right)}^{i+j}\prod_{k=j}^{i-1}\epsilon_{k}\sum_{m=j}^{i}\frac{e^{-t\epsilon_{m}}}{\left(\prod_{p=1}^{i-m}\epsilon_{m}\;-\;\epsilon_{m+p}\right)\left(\prod_{q=j}^{m\;-\;1}\epsilon_{m}-\epsilon_{q}\right)} & i\geq j \end{array} \end{array}$$ (5)

The essential element of formulas Eqs (3) to (5) from the standpoint of stochastic structure is the presence of high order products of the random variables *ϵ*_*m*_. When the *ϵ*_*m*_ are equal Eq (5) simplifies to the familiar Poisson:

$$\begin{array}{c} Q{\left(t\right)}_{i,j}=\{\begin{array}{cc} 0 & i<j\\ e^{-t\epsilon }\frac{{\left(t\epsilon \right)}^{i-j}}{(i-j)!} & i\geq j \end{array} \end{array}$$ (6)

Thus the discrete distribution *Q*(*t*)_*i*,*j*_ represents a generalization of the Poisson that incorporates multiplicative stochastic variation and is appropriate for the characterization of processes that involve discrete steps that are subject to inter-step variability. Both transcription and translation are such processes, although the focus of this work is translation. Translational variation is likely to be greater than transcriptional variation because of the greater variety of participating substrates and the larger number of discrete steps that must occur to effect the addition of a single residue to the elongating chain.

The structure of *Q*(*t*)_*i*,*j*_ can be rendered in the language of divided differences (Appendix A) as

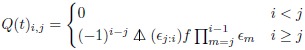
(7)
with *f*(*ϵ*) = *e*^−*tϵ*^. Thus the entries of *Q* are most simply the product of a divided difference of an exponential and a product of terms of the same order. Because a divided difference of order *n* is the discrete difference counterpart of a derivative of order *n*, we see two major contributions to stochastic instability: a product of random variables, and a high order difference function. It is well known numerically that instability in computation increases with the order of the difference/derivative.

## Steady state distribution of proteins

**Fig 2:**
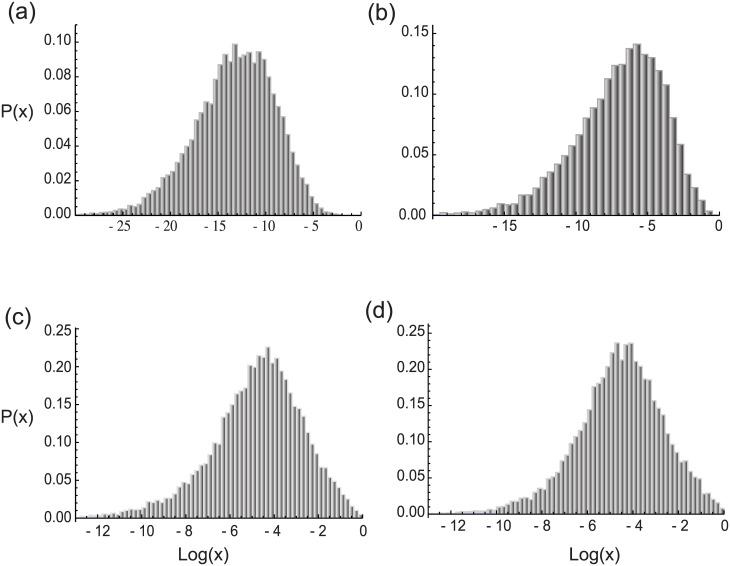
Simulation results for the distribution of proteins across cell population as the system evolves from an initial transient state and finally reaches the steady state. The plots give the distributions at times (a) *t* = 0.5*T*, (b) *t* = *T*, (c) *t* = 2*T* and (d) *t* = 3*T*, with *T* is an arbitrary unit of time. The distribution narrows between time 0.5*T* and time *T* while the dynamics are in the transient phase. The narrowing continues as the dynamics near steady state at time 2*T*. At time 3*T* a steady state distribution given by an approximate log-normal is reached, confirmed by the finding that for any further increase in time the distribution remains invariant and log-normal. In the simulation ribosome recycling is incorporated via circular boundary condition. The distribution of completed proteins is calculated by taking the difference of fluxes between the termination and initiation sites.

To explore the behavior of the distribution above, numerical simulations were performed for the translation of mRNAs incorporating a ribosome recycling step using the circular boundary condition on a transcript containing a ribosome at the origin, *V*(0) = {1, 0, 0 …, 0} at *t* = 0. The distribution of proteins was obtained as the difference in fluxes between the termination and re-initiation sites for increasing time (Fig 2(a)–2(c)), until such time as the steady state distribution of proteins has been reached (Fig 2(d)). The form of the protein distribution remains unchanged with any further increase in time, consistent with the asymptotic nature of this distribution.

The numerically obtained shape of the steady state distributions in Fig 2 resembles a log-normal distribution, which can be explained from the stochastic model outlined in the previous section.

The matrix action *V*(*t*)_*i*_ = *Q*(*t*)_*i*,*j*_*V*(0)_*j*_ on a pure initiation state at *t* = 0, (i.e. *V*(0) = {1, 0, 0 …, 0}), results in elements of *V*(*t*)_*i*_ determined entirely by *Q*(*t*)_*i*,1_, which is given by Eq (4) for the circular mRNAs and Eq (5) for the short lived mRNAs. In both these cases *Q*(*t*)_*i*,1_ is a sum of products of random variables. If the second moment of the logarithm of such variables is finite, the probability density of a product of random variables approaches a log-normal density as the number of variables grows large, and in such limit the probability density of *Q*(*t*)_*i*,*j*_ represents a sum of log-normal densities. A general analytical form for the density of a sum of products of random variables cannot be presented as the characteristic function does not have a closed form [54]. But numerical and analytical studies, particularly in the context of wireless communication and related fields, in which log-normal sums appear frequently, have shown that the sum of log-normal distributions has similar character to a log-normal distribution [55–57]. Consistent with this, the predicted consequences of translational rate variation give rise to approximately log-normal densities, as shown in Fig 2. The insensitivity of the density to summation for relatively large numbers of sums suggest that the densities of proteins in single cells when viewed as individual events contributing to a composite distribution should also appear approximately log-normal in the steady state.

The densities of sums of log-normally distributed variables are considered to be reasonably well described by another log-normal except in the tails of the density [55]. Empirical fits of simulation data to analytical distributions using the Mathematica FindDistribution function show that the probablility densities produced by the simulations calculated here can be fit with varying degrees of success to a sum of two log-normals or a sum of a log-logistic and a log-normal distribution.

**Fig 3:**
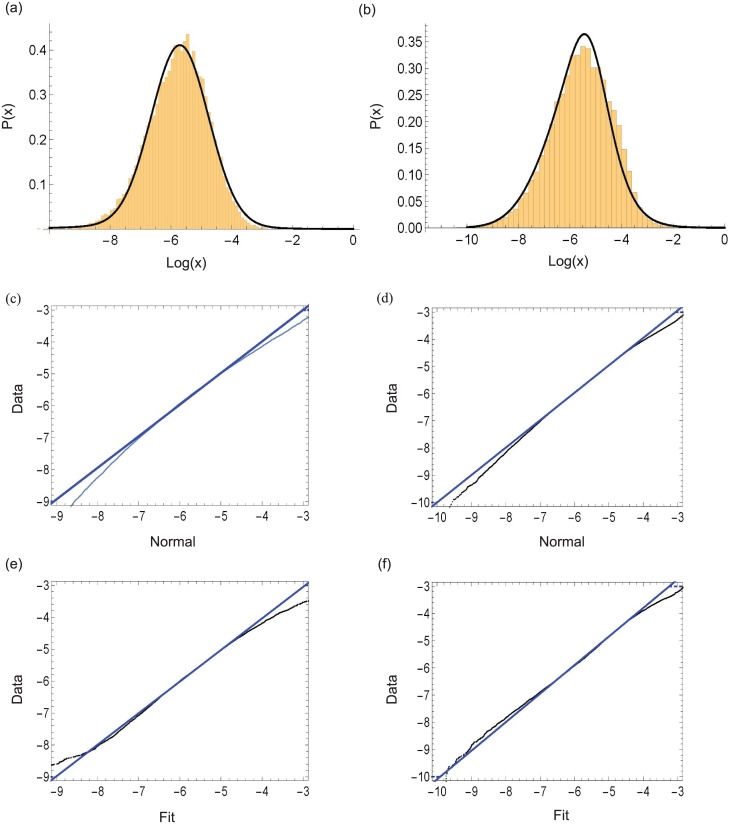
A quantile-quantile (Q-Q) plot shows that the steady state protein distribution is well described by a log-normal except in the tail region where deviations arise. The histograms (a) and (b) show the simulated results for the steady state protein distributions in cases in which the *ϵ*_*i*_ follow: (a) a gamma distribution (shape parameter 10, scale parameter 5); or (b) a normal distribution (mean 50 and standard deviation 15). The solid (black) line in (a) and (b) represent the best unbiased fit for the corresponding data. The best unbiased fit for (a) is a sum of two log-normal distributions with weights 0.985935 and 0.0140649, and for (b) is a sum of log-normal and log-logistic distributions with weights 0.368091 and 0.631909 respectively. In the (Q-Q) plots (c) and (d), the log-transformed steady state distributions data corresponding to (a) and (b) respectively are plotted against a normal distribution. In the (Q-Q) plots (e) and (f), the log-transformed steady state distributions data corresponding to (a) and (b) respectively are plotted against the best unbiased fit distribution. The relative improvement of fit compared to (c) and (d) is noticeable.

A convenient way to visualize the deviation of experimental data from a predicted distribution is the quantile-quantile plot (Q-Q plot). The quantiles of the simulation distribution are plotted against the test distribution quantiles which lie along *y* = *x* line. As can be seen in Fig 3, the log-transformed distributions exhibit normality over a wide range as its quantiles also lie on the *y* = *x* line, with deviations seen in the tails. The deviations in the tails of the distribution in our model indicate that protein abundance distributions in vivo may possess a larger dynamic range than log-normal distributions. These deviations may pose special challenges for cellular regulation if not accompanied by rapid regressions to the mean. In a multicellular organism, cells that are outliers with respect to expression may serve a protective or sentinel function, providing population responses that may have a greater dynamic range attributable to the response characteristics of outliers.

Alternate and somewhat more familiar distributions, such as the gamma distribution, have been proposed to account for protein abundance variation. Within the context of the model presented here, it should be clear that the essential feature of the variation arises from well-justified kinetic equations that indicate the productivity of a given transcript depends on an expression that contains high powers of products of random variables. As such, the theoretical starting point in the search for an appropriate model distribution should incorporate the ability to represent products, as opposed to sums, of random variables.

Broadly, our simulation results are consistent with the emergence of log-normality whenever the range of rate distributions for individual elongation steps remains as large between cells as between individual transcripts within the same cell. It is difficult to plausibly formulate circumstances under which this would not be true.

**Fig 4:**
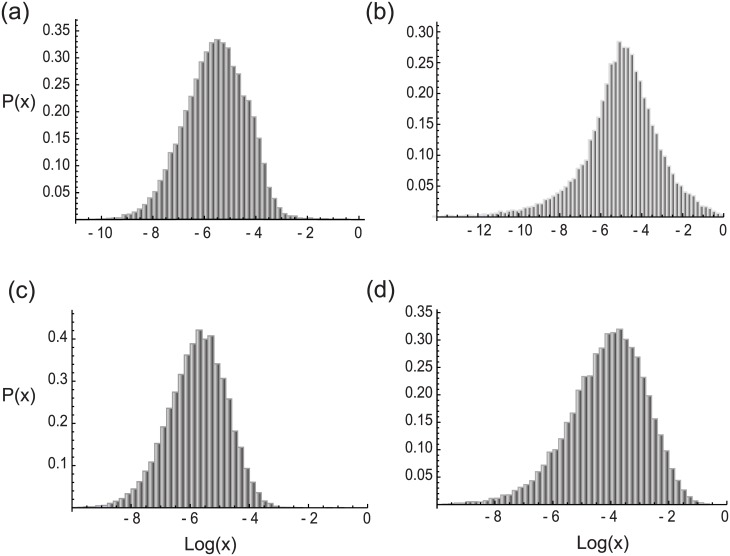
The steady state distribution of proteins does not depend on the precise form of the underlying rate constant distributions for elongation. The plots show the simulated results for the steady state protein distributions in cases in which the underlying rate constant distributions along the entire chain follow (a) normal distribution (mean 50 and standard deviation 15), (b) exponential distribution (mean 100), (c) gamma distribution (shape parameter 10, scale parameter 5), and (d) log-normal distribution (derived from normal distribution with mean 3.5 and standard deviation 1). The parameters of the scaled rate constant (*ϵ*) distribution were chosen so that the system is in the steady state phase in all plots.

An essential tenet of the law of large numbers is the independence of the limiting distribution of sums of variables upon the distributions of the individual variables [58]. Currently, accurate experimental data are not available for rate constant distributions in vivo. Based on chemical reaction kinetics a case can be made that the rate constant distribution may often have the exponential form of the typical Arrhenius distribution [59]. However, as living cells exist in conditions that are far from equilibrium and subject to regulatory influences the possibility that rate constant distributions assume some other form cannot be ruled out. In numerical simulations we have found that the steady state protein distribution form is largely unaffected by the changes in distribution of rate constants (*ϵ*_*i*_), as shown in Fig 4.

**Fig 5:**
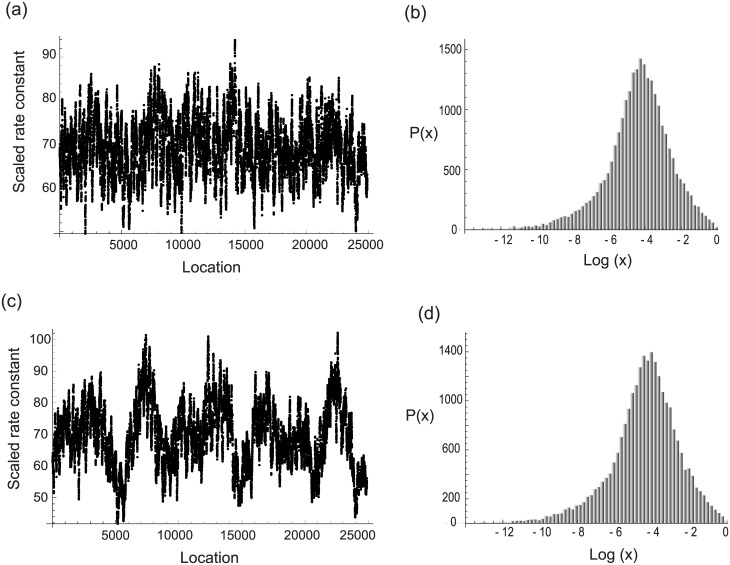
The incorporation of correlation between individual rates has little effect on the resulting steady state distribution. (a) The spatial arrangement of rate constants at an arbitrarily chosen mRNA site across an ensemble of mRNA molecules encoding the same protein. For visual clarity, the moving averages of rate constants along 100 adjacent mRNAs is shown. (b) Steady state distribution corresponding to (a). (c) Spatially correlated rate constants at an arbitrarily chosen site of mRNA (moving average over 100 sites is plotted) across an ensemble of mRNA. (d) Steady state distribution corresponding to (b).

The rate variables (*ϵ*_*i*_), which represent the rates for migration of the ribosome along the mRNA, are primarily influenced by factors that can be collectively described as extrinsic noise. Changes in protein abundance will contain deterministic components under conditions in which a cell or population is transiting the cell cycle, acclimating to environmental conditions, or undergoing differentiation [60]. The influence of deterministic components may produce correlations that affect the rate constants at any given position in the mRNA. However, the presence of correlation among rate constants does not significantly influence the steady state distribution, as shown in Fig 5.

The interaction between successively translated ribosomes such as collisions [46] will cause a reduction of the elongation rates for trailing ribosomes near the collision sites. The effect of the presence of codons with small values for *ϵ*_*i*_ (“slow codons”), either because their cognate tRNAs are underrepresented or because of specific contexts, such as secondary structure in the mRNA, that impede translation, may also cause similar lowering of elongation rate. These circumstances, in the extreme case, should have an effect that is equivalent to a rate limiting phase in the elongation cycle.

**Fig 6:**
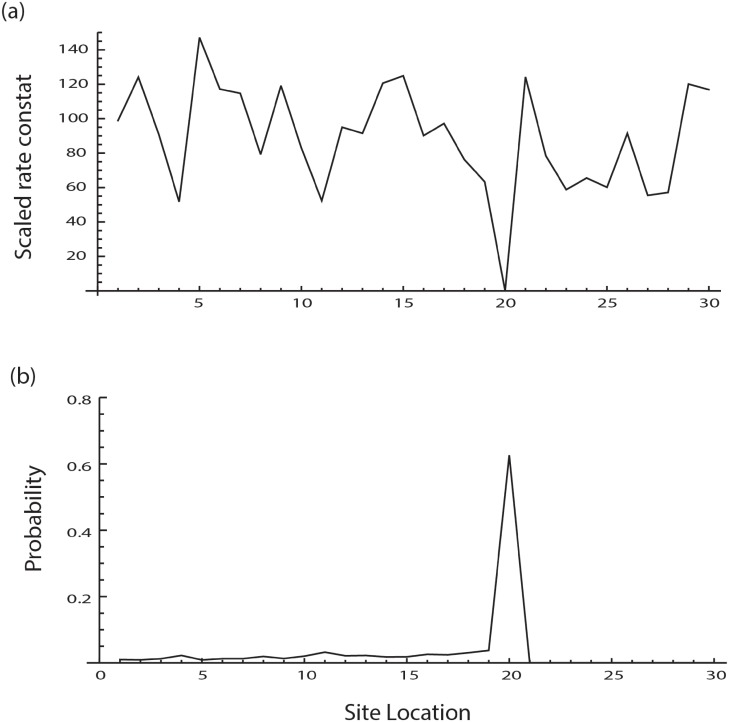
The effect of a low rate constant for elongation at a given residue. In this case it is expected that the elongation will stall at a site with a very low rate constant. (a) In order to simulate this condition the mean value for the rate constant distributions at various sites is allowed to vary between 50 and 150 except at site 20, where the mean value is set near zero. (b) The resulting distribution of the polypeptides across various sites clearly shows the stalling effect with resulting accumulation around the residue site 20. Although this example has been constructed to verify the validity of the model, the occurrence of pause sites has been reported in the literature.

To simulate this scenario a linear lattice of size 30 was chosen. At site 20 the scaled rate constant was set close to zero to represent the dynamical effects caused by the presence of a slow codon at that site. The calculation of the protein probability density in this condition confirms a local maximum around site 20, consistent with the expectation that the presence of a slow codon on mRNA and ribosome collison pauses translation leading to accumulation near the site (Fig 6). The results are similar when the ribosome collision sites and sites with slow codons are chosen near any arbitrary set of consecutive sites on the lattice. Such pausing and stacking effect has been reported in many different experiments, e.g. by Wolin and Walters [44].

## Distribution of the number of polypeptides per transcript

**Fig 7:**
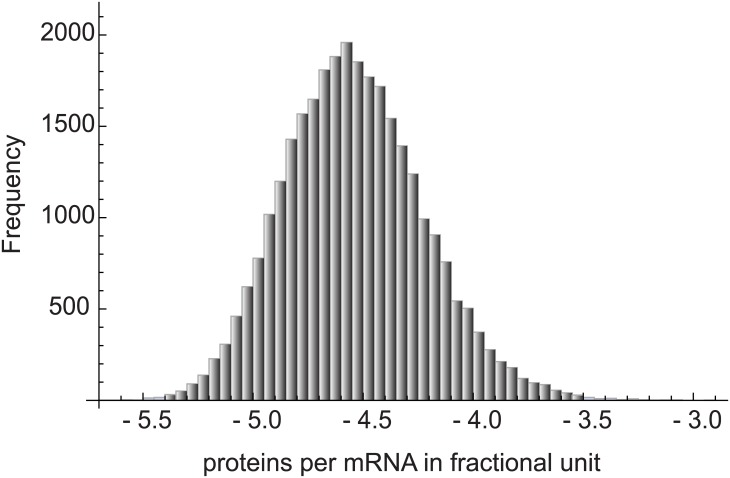
The number of proteins per mRNA follows a log-normal distribution. The plot shows the histogram of the protein abundance (in fractional units) per template. For the simulation of this plot, the underlying rate constant (*ϵ*) distribution is taken to follow a gamma distribution with shape parameter 10 and scale parameter 5. Similar results are obtained in cases in which the underlying rate constant distributions are different.

The number of polypeptides per transcript can be calculated with some assumptions about the decay kinetics of the transcripts. The rate of production of a completed polypeptide of length *d* is given by *ϵ*_*d*_*V*_*d*_(*t*) and the integral with respect to time over the lifetime of the transcript gives the number of polypeptides. For first order decay of transcripts with rate constant *c*, the distribution representing the location of the leading ribosome will be the Laplace transform in time of the transition operators with respect to conjugate variable *c*. Taking the example of Eq (4), the *i*, *j* entry of the matrix for *i* ≥ *j* gives

$$\begin{array}{c} Q{\left(t\right)}_{i,j}=\frac{\prod_{k=1}^{j-1}\left(c+\epsilon_{k}\right)\prod_{k=i+1}^{d}\left(c+\epsilon_{k}\right)\prod_{m=j}^{i-1}\epsilon_{m}}{\prod_{k=1}^{d}\left(c+\epsilon_{k}\right)-\lambda \prod_{m=1}^{d}\epsilon_{m}} \end{array}$$ (8)

with *d* the number of codons as in Eq (4). The structure of (6) shows the characteristic multiplicative interactions that contribute in an important way to the overall stochastic variation. The consequences of this multiplicative effect can be seen in Fig 7 which shows that the number of polypeptides per transcript calculated via simulation of Eq (8) produce a distribution with approximate log-normality.

## The transient phase of translation dynamics

**Fig 8:**
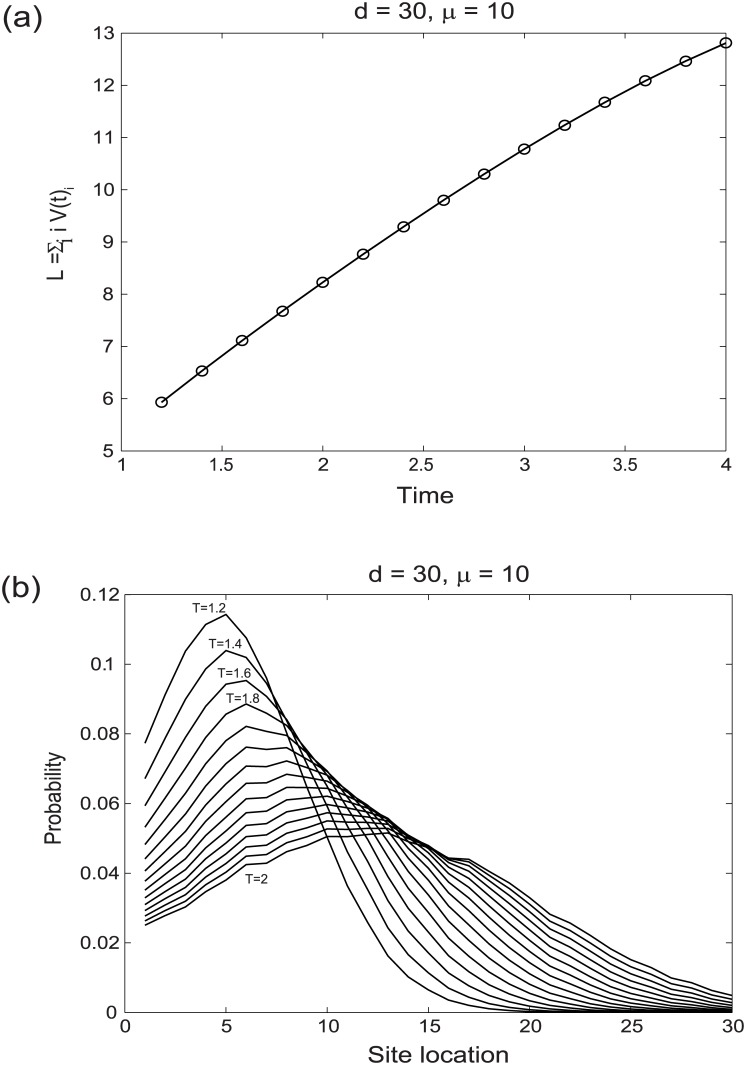
The dynamics of polypeptide chain formation. (a) The average length of polypeptide chain d increases linearly with time. (b) With increasing time the overall occupation probability of polypeptides decreases near the initiation site and increases near the termination site. L denotes length of the lattice chain and m is the mean value of the underlying rate constant distribution, which is given by an exponential distribution for this figure.

This section examines the effect of stochastic dynamics on a collection of mRNAs arising from a transcriptional burst in a cell population. One useful quantity that quantifies the transient phase of the dynamics is the average extent of polypeptide chain formation in a cell population. Let *L* be such a quantity described by the expectation value

$$\begin{array}{c} L=\sum_{i=1}^{d}iV_{i}\left(t\right) \end{array}$$ (9)

where *i* denotes the discrete site location and *V*_*i*_(*t*) gives the probability that elongation has proceeded up to site *i* at time *t* assuming the polypeptide has been initiated at time 0. Fig 8(a), is a plot of *L* for a lattice chain of length *d* = 30 with exponentially distributed rate constants of scaled mean 10. The expected length of the polypeptide chain formed in the cell population, *L*, varies linearly with time, except for a slight deviation from linearity at longer times. To mimic the physiological process, in the simulation fully formed proteins are required to decay with some probability. The mass of protein that decays after termination is proportional to the total amount of fully formed protein. The protein decay feature causes a deviation from linearity for *L* at longer times. Examination of the sum of site-specific probabilities across the cell population at increasing times (Fig 8(b)) reveals the effect of elongation proceeding towards the termination site. As expected, with time the occupation probability decreases near the initiation site and increases near the termination site as nascent peptides are released as fully formed proteins.

**Fig 9:**
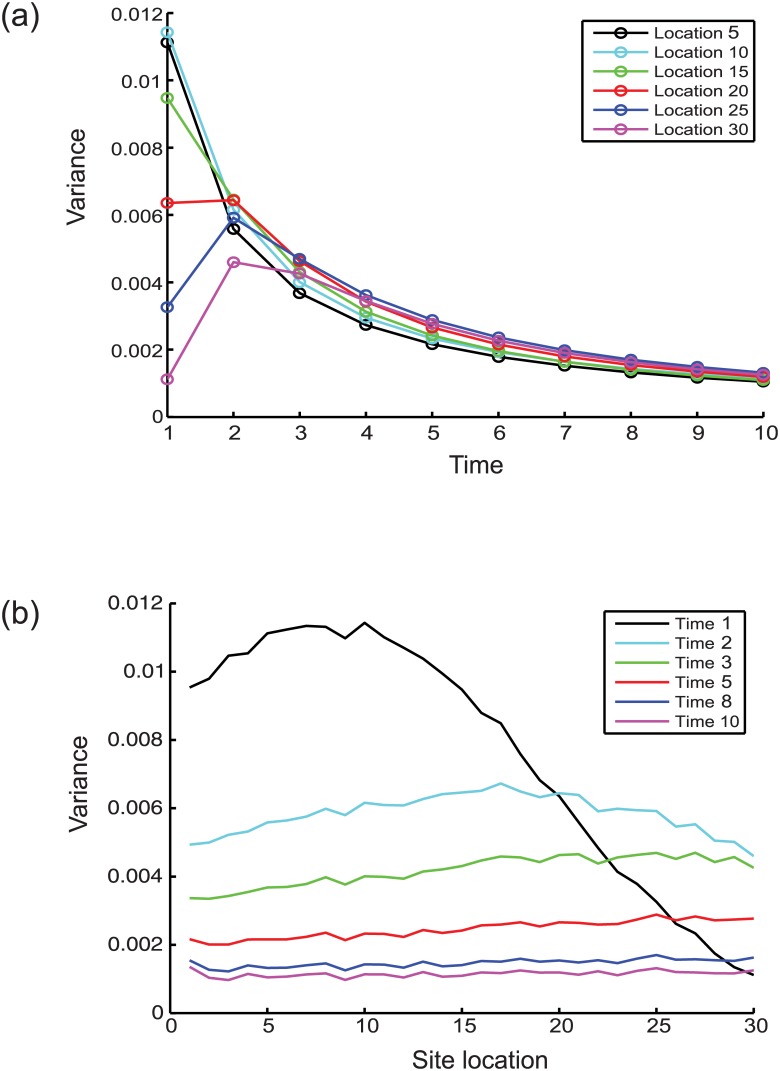
The change in estimated variance of the occupation probability at different sites along the mRNA. (a) The variation of the estimated variance at different residues along the mRNA chain with time. (b) The changes in estimated variance at different times along the mRNA chain at different residue locations. The estimated variance is obtained by taking the log-transform of a log-normal distribution and calculating the variance of the resulting normal distribution.

The variance of the occupation probabilities at different sites across a cell population along the mRNA chain is expected to change with time, as should the occupation probability variance at a particular site. In order to capture the nature of this variation the process was simulated with exponentially distributed spatially varying rate constants (*ϵ*_*i*_) over a linear chain of length 30 (Fig 9). With increasing time the numerically estimated variance at different locations tends to converge (Fig 9(a)) and decrease (Fig 9(b)).

## Conclusion

This work draws attention to the role played by translation rate fluctuation in determining the shape of steady state protein concentration distributions. The multiplicative sources of noise resulting from variations in the ribosomal translocation rates give rise to an approximately log-normal distribution. Physical and chemical laws that are dominated by multiplicative components rather than additive, are expected to lead to log-normality in natural systems [61]. The analysis presented here implies that the equilibrium protein concentrations across cell population are distributed so that the central part of the distribution follows a log-normal. This result is consistent with experimentally observed steady state protein distributions.

The tails of the distribution deviate from log-normal behavior. The tail characteristics are not robust and shows a variety a behaviors depending on the form of the underlying rate constant distributions (Fig 3). How the dynamical influences of rate constant fluctuations modulate the behavior in the tail of the steady state protein distribution requires further study.

The model described here can be made more specific by explicitly incorporating additional factors that influence protein concentrations, such as transcription or factors involved in protein degradation. But in its current form, the model provides an explanation for the experimentally observed log-normal protein distribution across cell population and confirms the conclusion drawn by several recent studies that indicate that protein abundance is primarily determined by extrinsic noise sources which control translation.

A general framework for characterizing the transition matrices for stepwise processes that relies on the properties of polynomial functions of a complex variable has been provided. This framework offers a consistent general approach for the derivation of stochastic group and semigroup operators and supports the identification of the transition process with random step probability as a natural generalization of the Poisson to processes with multiplicative noise.

Considerable effort has been devoted to characterizing various aspects of the origin of stochastic gene expression in cellular processes [29, 36]. This work draws attention to the significant and likely dominant role played by translational noise in stochastic gene expression in living cells. Progress in single cell measurements of translational parameters will undoubtedly enhance our understanding of the sources of variation in gene expression.

## Appendix A

### Contour integral form for matrix power series

In eukaryotic cells evidence of mRNA template circularization has been observed, both biochemically in the form of protein complexes that bind to both poly(A) and the mRNA cap structure [62], and ultrastructurally, in the form of polysomes linked in circular configuration. To model the motion of ribosomes on such a template, we identify the generator for the translation operator with the structure

$$\begin{array}{c} T=(\begin{array}{ccccc} -\epsilon_{1} & 0 & \cdots & 0 & \lambda \;\epsilon_{d}\\ \epsilon_{1} & -\epsilon_{2} & \cdots & 0 & 0\\ \vdots & \vdots & \ddots & \vdots & \vdots \\ 0 & 0 & \cdots & -\epsilon_{d-1} & 0\\ 0 & 0 & \cdots & \epsilon_{d-1} & -\epsilon_{d} \end{array}) \end{array}$$ (10)

from which we construct the desired solution as the sum $\sum_{n=0}^{\infty }{\left(tT\right)}^{n}/n!$. A consistent and general representation of the terms of the power series expansion of *T*^*k*^ = (*U* − 1)^*k*^ can be obtained in the form of a contour integral. In general the matrix *T*(*d*)^*k*^ with elements *i*, *j* can be represented by an integral

$$\begin{array}{c} T{\left(d\right)}_{i,j}^{k}=\frac{1}{2\pi ı}\oint g_{i,j}\left(s,d\right)s^{k}ds \end{array}$$ (11)

where *g*_*i*,*j*_(*s*, *d*) is a rational function (i.e. a function $f\left(s\right)=\frac{p(s)}{q(s)}$, *p*, *q* both polynomials of finite order). To avoid confusion of terms with the residues of a protein, the residue of the complex function *f* will be referred to as the “residue of integration,” in keeping with the interpretation that the contour integral along a path enclosing a function analytic within the contour is zero, whereas if the function has a pole within the contour there is a residual value resulting from integration. For integrals of such functions, the sum of the residue of integration of *f*(*s*) at infinity plus the sum of the residues of integration at the zeroes of *q* equals zero. It is convenient for the purpose of proof by induction to transform the integrand, recalling that the residue of integration at infinity of *f*(*s*) is defined as the negative of the residue of integration at 0 of *z*^−2^ *f*(1/*z*), where *z* = *s*^−1^.

The *i*, *j* entry of the *n*^*th*^ power of this matrix representing the sum $\sum_{n=0}^{\infty }{\left(tT\right)}^{n}/n!$ takes the form, for *i* < *j*,

$$\begin{array}{c} \frac{1}{2\pi ı}\oint \frac{\lambda \prod_{k=i+1}^{j-1}\left(1+z\epsilon_{k}\right)\prod_{m=j}^{d}\epsilon_{m}\prod_{m=1}^{i-1}\epsilon_{m}}{z^{j-i+n+1-d}\left(\prod_{k=1}^{d}\left(1+z\epsilon_{k}\right)-\lambda z^{d}\prod_{m=1}^{d}\epsilon_{m}\right)}dz \end{array}$$ (12)

and

$$\begin{array}{c} \frac{1}{2\pi ı}\oint \frac{\prod_{k=1}^{j-1}\left(1+z\epsilon_{k}\right)\prod_{k=i+1}^{d}\left(1+z\epsilon_{k}\right)\prod_{m=j}^{i-1}\epsilon_{m}}{z^{j-i+n+1}\left(\prod_{k=1}^{d}\left(1+z\epsilon_{k}\right)-\lambda z^{d}\prod_{m=1}^{d}\epsilon_{m}\right)}dz \end{array}$$ (13)

for *i* ≥ *j*, where the contour of integration encloses the origin but none of the roots of the denominator polynomial $\prod_{k=1}^{d}\left(1+z\epsilon_{k}\right)-\lambda z^{d}\prod_{m=1}^{d}\epsilon_{m}$. To establish this by induction we first observe that the variable *n* appears only in the denominator in the exponent of *z*. To calculate the residues of integration at zero one must consider five cases for *n* = 1: (i) *i* = 1, *j* = *d*; (ii) *i* < *j* (excepting case (i)); (iii) *i* = *j*; (iv) *i* = *j* + 1; and (v) *i* > *j* + 1. For case (i), *z*^*j*−*i*+2−*d*^ = *z* and the limit of Eq (12) as *z* → 0 is

$$\begin{array}{c} \frac{1}{2\pi ı}\oint \frac{\lambda \epsilon_{d}}{z\left(1+z\epsilon_{1}\right)\left(1+z\epsilon_{d}\right)}dz=\lambda \epsilon_{d} \end{array}$$ (14)

For case (ii), the limit of Eq (12) as *z* → 0 is dominated by *j* − *i* + 2 − *d* ≤ 0 and the function is analytic within the contour:

$$\begin{array}{c} \frac{1}{2\pi ı}\oint \frac{\lambda \prod_{m=j}^{d}\epsilon_{m}\prod_{m=1}^{i-1}\epsilon_{m}}{z^{j-i+2-d}}dz=0 \end{array}$$ (15)

For case (iii) the limit of Eq (13) as *z* → 0 is

$$\begin{array}{c} \frac{1}{2\pi ı}\oint \frac{1}{z^{2}\left(1+z\epsilon_{i}\right)}dz\to \frac{1}{2\pi ı}\oint (\frac{1}{z^{2}}-\frac{\epsilon_{i}}{z})dz=-\epsilon_{i} \end{array}$$ (16)

For case (iv) the limit of Eq (13) as *z* → 0 is

$$\begin{array}{c} \frac{1}{2\pi ı}\oint \frac{\epsilon_{j}}{z\left(1+z\epsilon_{j}\right)\left(1+z\epsilon_{j+1}\right)}dz=\epsilon_{j} \end{array}$$ (17)

and for case (v) the limit of Eq (13) as *z* → 0 is dominated by *j* − *i* + 2 ≤ 0 and the function is analytic within the contour:

$$\begin{array}{c} \frac{1}{2\pi ı}\oint \frac{\prod_{k=j}^{i-1}\epsilon_{k}}{z^{j-i+2}}dz=0 \end{array}$$ (18)

To complete a proof by induction one must formally establish, using Eqs (10)–(13) that *TT*^*n*^ = *T*^*n*+1^. The actual process is slightly different; we establish that

$$\begin{array}{c} TT^{n}-T^{n+1}=-\frac{1}{2\pi ı}\oint 1_{d}z^{-n-2}dz=0_{d} \end{array}$$ (19)

where $1_{d}$ and $0_{d}$ are the *d* dimensional unit and null matrices, respectively. There are 5 cases to be calculated: (i) *i* < *j* (*i* = 1); (ii) *i* < *j* (*i* > 1); (iii) *i* = *j* (*i* = 1); (iv) *i* = *j* (*i* > 1); and (v) *i* > *j*. For case (i) we establish

$$\begin{array}{l} \lambda \epsilon_{d}\frac{\prod_{k=1}^{j-1}\left(1+z\epsilon_{k}\right)\prod_{m=j}^{d-1}\epsilon_{m}}{z^{j-d+n+1}\left(\prod_{k=1}^{d}\left(1+z\epsilon_{k}\right)-\lambda z^{d}\prod_{m=1}^{d}\epsilon_{m}\right)}\\ -\left(z^{-1}+\epsilon_{1}\right)\frac{\prod_{k=2}^{j-1}\left(1+z\epsilon_{k}\right)\lambda \prod_{m=j}^{d}\epsilon_{m}}{z^{j-d+n}\left(\prod_{k=1}^{d}\left(1+z\epsilon_{k}\right)-\lambda z^{d}\prod_{m=1}^{d}\epsilon_{m}\right)}=0 \end{array}$$ (20)

For case (ii),

$$\begin{array}{c} \epsilon_{i-1}\frac{\lambda \prod_{k=i}^{j-1}\left(1+z\epsilon_{k}\right)\prod_{m=j}^{d}\epsilon_{m}\prod_{m=1}^{i-2}\epsilon_{m}}{z^{j-i+n+2-d}(\prod_{k=1}^{d}\left(1+z\epsilon_{k}\right)-\lambda z^{d}\prod_{m=1}^{d}\epsilon_{m})}\\ -\left(z^{-1}+\epsilon_{i}\right)\frac{\prod_{k=i+1}^{j-1}\left(1+z\epsilon_{k}\right)\lambda \prod_{m=j}^{d}\epsilon_{m}\prod_{m=1}^{i-1}\epsilon_{m}}{z^{j-i+n+1-d}(\prod_{k=1}^{d}\left(1+z\epsilon_{k}\right)-\lambda z^{d}\prod_{m=1}^{d}\epsilon_{m})}=0 \end{array}$$ (21)

For case (iii)

$$\begin{array}{c} \lambda \epsilon_{d}\frac{\prod_{m=1}^{d-1}\epsilon_{m}}{z^{n-d+2}(\prod_{k=1}^{d}\left(1+z\epsilon_{k}\right)-\lambda z^{d}\prod_{m=1}^{d}\epsilon_{m})}\\ -\left(z^{-1}+\epsilon_{i}\right)\frac{\prod_{k=2}^{d}\left(1+z\epsilon_{k}\right)}{z^{n+1}(\prod_{k=1}^{d}\left(1+z\epsilon_{k}\right)-\lambda z^{d}\prod_{m=1}^{d}\epsilon_{m})}=-z^{-n-2} \end{array}$$ (22)

For case (iv)

$$\begin{array}{c} \epsilon_{i-1}\frac{\lambda \prod_{k=i}^{j-1}\left(1+z\epsilon_{k}\right)\prod_{m=j}^{d}\epsilon_{m}\prod_{m=1}^{i-2}\epsilon_{m}}{z^{j-i+n+2-d}(\prod_{k=1}^{d}\left(1+z\epsilon_{k}\right)-\lambda z^{d}\prod_{m=1}^{d}\epsilon_{m})}\\ -\left(z^{-1}+\epsilon_{i}\right)\frac{\prod_{k=1}^{j-1}\left(1+z\epsilon_{k}\right)\prod_{k=i+1}^{d}\left(1+z\epsilon_{k}\right)\prod_{k=j}^{i-1}\epsilon_{m}}{z^{j-i+n+1}(\prod_{k=1}^{d}\left(1+z\epsilon_{k}\right)-\lambda z^{d}\prod_{m=1}^{d}\epsilon_{m})}=-z^{-n-2} \end{array}$$ (23)

And for case (v)

$$\begin{array}{c} \epsilon_{i-1}\frac{\prod_{k=1}^{j-1}\left(1+z\epsilon_{k}\right)\prod_{k=i}^{d}\left(1+z\epsilon_{k}\right)\prod_{m=j}^{i-2}\epsilon_{m}}{z^{j-i+n+2}(\prod_{k=1}^{d}\left(1+z\epsilon_{k}\right)-\lambda z^{d}\prod_{m=1}^{d}\epsilon_{m})}\\ -\left(z^{-1}+\epsilon_{i}\right)\frac{\prod_{k=1}^{j-1}\left(1+z\epsilon_{k}\right)\prod_{k=i+1}^{d}\left(1+z\epsilon_{k}\right)\prod_{m=j}^{i-1}\epsilon_{m}}{z^{j-i+n+1}(\prod_{k=1}^{d}\left(1+z\epsilon_{k}\right)-\lambda z^{d}\prod_{m=1}^{d}\epsilon_{m})}=0 \end{array}$$ (24)

A more convenient characterization of Eqs (12) and (13) for evaluation of the steady state formulates the matrix in terms of *s* = *z*^−1^ and gives, for the *i*, *j* entry of the *n*^*th*^ power of the matrix Eq (11), for *i* < *j*

$$\begin{array}{c} \frac{1}{2\pi ı}\oint \frac{s^{n}\lambda \prod_{k=i+1}^{j-1}\left(s+\epsilon_{k}\right)\prod_{m=j}^{d}\epsilon_{m}\prod_{m=1}^{i-1}\epsilon_{m}}{\prod_{k=1}^{d}\left(s+\epsilon_{k}\right)-\lambda \prod_{m=1}^{d}\epsilon_{m}}ds \end{array}$$ (25)

and

$$\begin{array}{c} \frac{1}{2\pi ı}\oint \frac{s^{n}\prod_{k=1}^{j-1}\left(s+\epsilon_{k}\right)\prod_{k=i+1}^{d}\left(s+\epsilon_{k}\right)\prod_{m=j}^{i-1}\epsilon_{m}}{\prod_{k=1}^{d}\left(s+\epsilon_{k}\right)-\lambda \prod_{m=1}^{d}\epsilon_{m}}ds \end{array}$$ (26)

otherwise, where the contour encloses all of the roots of the denominator polynomial $\prod_{k=1}^{d}\left(s+\epsilon_{k}\right)-\lambda \prod_{m=1}^{d}\epsilon_{m}$. For λ = 1, *T* is a stochastic (conservative) matrix, and therefore should have a steady state given by the residues of integration of Eqs (25) and (26) at *s* = 0. Inspection of Eqs (25) and (26) shows that for λ = 1, *s* = 0 is indeed a root of the denominator polynomial, and hence the steady state solution is given by the residue of integration at 0 of the sum of Eqs (25) and (26) over *n* with *s*^*n*^ replaced by (*ts*)^*n*^/*n*!, which is

$$\begin{array}{c} \frac{\prod_{m=1}^{d}\epsilon_{m}}{\epsilon_{i}\sum_{k=1}^{d}\frac{\prod_{m=1}^{d}\epsilon_{m}}{\epsilon_{k}}}=\frac{1}{\epsilon_{i}\sum_{k=1}^{d}\frac{1}{\epsilon_{k}}} \end{array}$$ (27)

in both cases. The *i*, *j* entry of the matrix representing the steady state distribution has no dependence on *j*, consistent with intuition.

In the case λ = 0, the *i*, *j* entry of the *n*^*th*^ power of matrix (10) is zero for *i* < *j*, and

$$\begin{array}{c} \frac{1}{2\pi ı}\oint \frac{s^{n}\prod_{m=j}^{i-1}\epsilon_{m}}{\prod_{k=j}^{i}\left(s+\epsilon_{k}\right)}ds \end{array}$$ (28)

otherwise, where the contour taken in the conventional (positive) sense encloses all of the roots of the denominator polynomial (i.e. encircles all of the real axis values of −*ϵ*_*k*_, *j* ≤ *k* ≤ *i*. The matrix *e*^*tT*^ in this case is given by Eq (28) with *s*^*n*^ replaced by the sum $\sum_{n=0}^{\infty }{\left(ts\right)}^{n}/n!$, the integral of which converges, despite its resemblance to a function with an essential singularity at infinity. The evaluation of the contour integral for *n* = 0 yields the unit matrix as required. Evaluation of the integral leads to (*e*^*tT*^)_*i*,*j*_ = 0 for *i* < *j*, and

$$\begin{array}{c} {\left(e^{tT}\right)}_{i,j}={\left(-1\right)}^{i+j}\sum_{k=j}^{i}\frac{e^{-t\epsilon_{k}}\prod_{m=j}^{i-1}\epsilon_{m}}{\prod_{p=1}^{i-k}\left(\epsilon_{k}-\epsilon_{k+p}\right)\prod_{q=j}^{k-1}\left(\epsilon_{k}-\epsilon_{q}\right)} \end{array}$$ (29)

otherwise.

In the event that all of the *ϵ*_*k*_ are equal, the evolution operator represented by Eq (29) takes the particularly simple form of a Poisson distribution

$$\begin{array}{c} \frac{{\left(t\epsilon \right)}^{i-j}}{(i-j)!}e^{-t\epsilon } \end{array}$$ (30)

for *i* ≥ *j* (and 0 otherwise) and hence Eq (29) can be considered a natural generalization of a Poisson process to a domain in which the underlying stochastic process is not homogeneous. The gamma distribution, an extension of the Poisson to nonintegral event frequencies, takes the related form

$$\begin{array}{c} \frac{{\left(t\epsilon \right)}^{k}}{\Gamma (k+1)}e^{-t\epsilon } \end{array}$$ (31)

which bears comparison to Eq (29) because Eq (31) has been proposed to appropriately capture the statistics of low multiplicity translations emitted by a single mRNA template.

When the operator *T* acts on an initial state vector *V*(*t*_0_) = 1, 0, …, 0 of length *d* at time *t*_0_ = 0, *e*^*tT*^*V*(0) gives the probability density of the location of a ribosome on the mRNA at time *t*. If the source of the translation is an mRNA with a probability of existence at time *t* of *c* *e*^−*ct*^, the relative effect of additional initiations will be given by $\int_{0}^{\infty }c\;e^{-ct}e^{tT}v\left(0\right)dt$, and the *i*, *j* entry of $\int_{0}^{\infty }c\;e^{-ct}e^{tT}dt$ for *i* < *j* is

$$\begin{array}{c} \frac{c\lambda \prod_{k=i+1}^{j-1}\left(c+\epsilon_{k}\right)\prod_{m=j}^{d}\epsilon_{m}\prod_{m=1}^{i-1}\epsilon_{m}}{\prod_{k=1}^{d}\left(c+\epsilon_{k}\right)-\lambda \prod_{m=1}^{d}\epsilon_{m}} \end{array}$$ (32)

and

$$\begin{array}{c} \frac{c\prod_{k=1}^{j-1}\left(c+\epsilon_{k}\right)\prod_{k=i+1}^{d}\left(c+\epsilon_{k}\right)\prod_{m=j}^{i-1}\epsilon_{m}}{\prod_{k=1}^{d}\left(c+\epsilon_{k}\right)-\lambda \prod_{m=1}^{d}\epsilon_{m}} \end{array}$$ (33)

otherwise.

The rate of production of full length protein is given by *v*(*t*)_*d*_*ϵ*_*d*_ = *e*^*tT*^ *V*(0)_*d*_*ϵ*_*d*_ = (*e*^*tT*^)_*d*,1_*ϵ*_*d*_ and the integral with respect to time weighted by the lifetime of the encoding RNA gives

$$\begin{array}{c} c\;\epsilon_{d}\int_{0}^{\infty }e^{-ct}{\left(e^{tT}\right)}_{d,1}dt=\frac{c\prod_{m=1}^{d}\epsilon_{m}}{\prod_{k=1}^{d}\left(c+\epsilon_{k}\right)-\lambda \prod_{m=1}^{d}\epsilon_{m}} \end{array}$$ (34)

for the average number of polypeptides produced per mRNA template, assuming that the characteristic lifetime of the mRNA, *c*^−1^, is long compared to the translation time. In the event this is not true, we can estimate the number of polypeptides per template in the elongation-limited domain by dividing the mean length that the lead ribosome has translated down the mRNA, divided by the average number of residues between successive ribosomes, *R*. This has the form

$$\begin{array}{c} \sum_{i=1}^{d}\frac{i}{R}\int_{0}^{\infty }ce^{-ct}v{\left(t\right)}_{i}dt=\sum_{i=1}^{d}\frac{i}{R}\int_{0}^{\infty }ce^{-ct}{\left(e^{tT}\right)}_{i,1}dt \end{array}$$ (35)

which, using Eq (32) and setting λ = 0 (since the probability of reinitiation can be neglected), gives

$$\begin{array}{c} \sum_{i=1}^{d}\frac{i}{R}\frac{c\prod_{m=1}^{i-1}\epsilon_{m}}{\prod_{k=1}^{i}\left(c+\epsilon_{k}\right)} \end{array}$$ (36)

for the mean number of ribosomes per template over the life of the template. In the limit that all *ϵ*_*k*_ are equal, Eq (34) gives

$$\begin{array}{ll} \sum_{i=1}^{d}\frac{i}{R}\frac{c}{\epsilon \;{\left(1+\frac{c}{\epsilon }\right)}^{i}} & =\frac{\left(c+\epsilon \right)\;\left(1-{\left(\frac{c+\epsilon }{\epsilon }\;\right)}^{-d}\right)\;-\;cd\;{\left(\frac{c+\epsilon }{\epsilon }\right)}^{-d}}{Rc}\\ & \approx \frac{\left(c+\epsilon \right)\;\left(1-e^{-\frac{cd}{\epsilon }}\right)\;-\;cd\;e^{-\frac{cd}{\epsilon }}}{Rc}\\ & \approx \frac{cd\left(d+1\right)}{2R\epsilon } \end{array}$$ (37)

the approximations holding for *c* ≪ *ϵ*.

### Connection with divided differences

Divided differences [63] are nested differences defined recursively by the sequence

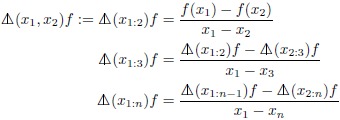
(38)
using a notation 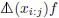 that is a variation [64] [63] on the representation introduced by Aitken [65]. In general

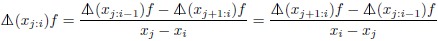
(39)

$$=\overset{i}{\underset{k=j}{\sum }}\frac{f(x_{k})}{\prod_{p=1}^{i-k}\left(x_{k}-x_{k+p}\right)\prod_{q=j}^{k-1}\left(x_{k}-x_{q}\right)}$$ (40)

With this expansion and *f*(*ϵ*) = *e*^−*tϵ*^ Eq (29) becomes (using (−1)^2*j*^ = 1)

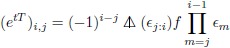
(41)
and the reduction to a Poisson Eq (30) reflects the fact that the divided difference has a limit for suitably differentiable functions of

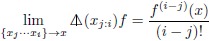
(42)
where *f*^(*n*)^(*x*) is the *n*th derivative with respect to x. A more direct connection can be made through the contour integral formula of Frobenius [63] which can be restated in our context as

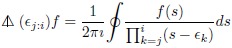
(43)
A Mathematica notebook for this appendix can be found at https://github.com/bs5975/Datta-Seed-Appendix-A.

## Appendix B

### Notes on numerical simulations

For numerical calculations, an ensemble of 25,000 *U* matrices were constructed for a chain of length *d* = 30. The matrices were populated with *ϵ* values drawn from a specific common distribution, such as an exponential or normal, with the *ϵ*_*i*_ varying from site to site. The time evolution of the extent of polypeptide formation was calculated by using Eq (1) in Section III. For the distribution of elongation rates, the rate constants at various sites were scaled by both *α* and time, where *α* has the dimension of inverse time. Note from Eq (1) that once *α* is fixed, longer time evolution is given by scalar multiplication of the rate constant distribution values by a larger factor. In general values were chosen to avoid crossing the bounds of numerical precision afforded by the machine and software architectures. In the rare cases that out-of-bound exceptions were thrown, the data points were discarded.

For shorter time evolution, the simulation results for the dynamics during the transient phase are described in Section VI. For longer time evolution, the extent of polypeptide formation calculated over an ensemble of 25,000 at the termination site is representative of the total quantities of proteins across cell population. Fig 2 shows the evolution of fully formed polypeptide distribution as the system passes through the transient phase to reach a statistically steady state characterized by an invariant probability density.

The best fit for the steady state distribution in Fig 3 was evaluated using the Mathematica function FindDistribution. The resulting fit is unbiased in the sense that no pre-specified target function was used to find the best fit.

The numerical simulations presented in the paper were carried out in 32 bit Matlab R2009b and Mathematica 11.1. Code can be found at https://github.com/sdatta91/PD-code.git.

## Appendix C

### Codon optimization and the rate-limiting step in translation

Consider the following step-wise procedure for codon optimization. For convenience we assume a circular template and individually measure each of the rate constants *ϵ*_*i*_. Let all of the *ϵ*_*i*_ be unequal. Then there is a smallest *ϵ*_*i*_ and that *ϵ*_*i*_ is limiting: it sets the rate at which the ribosomes circulate on the template. We then optimize that codon. Now there is a new location, *k*, at which *ϵ*_*k*_ is the smallest of the *ϵ*. We optimize that codon, and proceed. Eventually, we reach a point at which the rate-limiting codon cannot be substituted with another codon with an increase in the rate, and our optimization has come to an end. That we can achieve a similar result by changing all of the codons at once is not particularly relevant, except possibly as it affords additional improvements that may result from altering interactions between codons, for example when individually optimal codons form structures that impede translation.

When the rate-limiting codon that cannot be improved is the initiation codon, initiation is rate-limiting. But this situation cannot describe the initial state of the template, otherwise changing any of the other codons would have no effect.

In the foregoing the device of a circular template can be considered a heuristic aid. At steady state on a linear template, a ribosome released from the template takes a step on a circular lattice to position 0, representing the free ribosome pool.

## Appendix D

### List of symbols

$0_{d}$ the null *d* × *d* matrix
$1_{d}$ the unit *d* × *d* matrix
*I* the unit *d* × *d* matrix
*Q*(*t*) convolution semi-group operator (a *d* × *d* matrix)
*R* the average number of residues between successive ribosomes
*T* generator of the convolution semi-group (a *d* × *d* matrix)
*U* stochastic transition matrix of length *d*
*V*(*t*) vector representing the occupation state of *d* codons
*V*_*i*_(*t*) the *i*th entry of vector *V*(*t*)
*c* the rate constant of an mRNA undergoing first order decay
*d* number of codons in open reading frame
*f* any suitably smooth, e.g. *C*^∞^, function
*f*^(*k*)^(*x*) the *k*th derivative of *f*(*x*) with respect to x
*g*_*i*,*j*_(*s*, *d*) the *i*, *j*th entry of a matrix of rational functions of the complex variable *s*
*p*_*i*_ probability of transition from codon *i* to codon *i* + 1
*s* complex variable of integration
*t* time variable
*z* complex variable of integration
*α* proportionality between *p*_*i*_ and *ϵ*_*i*_ (*αp*_*i*_ = *ϵ*_*i*_)
*ϵ*_*i*_ rate of transition from codon *i* to codon *i* + 1
λ probability of reinitiation by a ribosome at termination codon
*ρ*(*p*_*i*_) probability density of the transition probability *p*_*i*_
ı $\sqrt{-1}$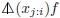 divided difference of *f* over the variables *x*_*j*_ ⋯ *x*_*i*_
